# Number of positive lymph nodes affects oncologic outcomes in cN0 mucoepidermoid carcinoma of the major salivary gland

**DOI:** 10.1038/s41598-024-59757-2

**Published:** 2024-04-20

**Authors:** Le Chang, Yingnan Wang, Zhen Wang, Di Xiao, Qi Song

**Affiliations:** 1 Department of Stomatology, The First Affiliated Hospital of Dalian Medical University, Dalian, Liaoning People’s Republic of China; 2 Stomatology Hospital, School of Stomatology, Zhejiang University School of Medicine, Zhejiang Provincial Clinical Research Center for Oral Diseases, Key Laboratory of Oral Biomedical Research of Zhejiang Province, Cancer Center of Zhejiang University, Engineering Research Center of Oral Biomaterials and Devices of Zhejiang Province CN, Hangzhou, People’s Republic of China

**Keywords:** SEER, Survival, Mucoepidermoid carcinoma, Major salivary gland, Prognostic model, Head and neck cancer, Oral cancer, Cancer, Surgical oncology

## Abstract

The survival significance of the number of positive lymph nodes in salivary gland carcinoma remains unclear. Thus, the current study aimed to determine the effect of the number of positive lymph nodes on disease-specific survival (DSS) and overall survival (OS) in cN0 mucoepidermoid carcinoma (MEC) of the major salivary gland. Patients surgically treated for MEC of the major salivary gland between 1975 and 2019 were retrospectively enrolled from the surveillance, epidemiology, and end results database. The total population was randomly divided into training and test groups (1:1). Primary outcome variables were DSS and OS. Prognostic models were constructed based on the independent prognostic factors determined using univariate and multivariate Cox analyses in the training group and were validated in the test group using C-index. A total of 3317 patients (1624 men and 1693 women) with a mean age of 55 ± 20 years were included. The number of positive lymph nodes was an independent prognostic factor for both DSS and OS, but the effect began when at least two positive lymph nodes for DSS and three positive lymph nodes for OS were found. Predictive models for DSS and OS in the training group had C-indexes of 0.873 (95% confidence interval [CI] 0.853–0.893) and 0.835 (95% CI 0.817–0.853), respectively. The validation of the test group showed C-indexes of 0.877 (95% CI 0.851–0.902) for DSS and 0.820 (95% CI 0.798–0.842) for OS. The number of positive lymph nodes was statistically associated with survival in cN0 major salivary gland MEC. The current prognostic model could provide individualized follow-up strategies for patients with high reliability.

## Introduction

Salivary gland carcinomas are relatively uncommon, accounting for approximately 1–3% of all head and neck tumors^1^. Most of these carcinomas occur in the major gland that consists of the parotid, submandibular, and sublingual glands^2^. Mucoepidermoid carcinoma (MEC) is the most frequent histologic type^3^. Surgery is the preferred treatment, but the value of elective neck dissection (END) remains controversial^4–6^. The rate of occult metastasis ranges from 2.2 to 26.4%^7–10^, and END is commonly recommend if this rate is estimated to be > 20%^11^. The possibility of occult metastasis is apparently influenced by high grade, advanced tumor stage, intraparotid lymph node metastasis, perineural invasion, and lymphovascular invasion^12^. Whether other potential predictors exist remains unknown.

Lymph node status is one of the most important prognostic factors^13^; the presence of only one positive lymph node decreases survival in many solid malignancies^2,3^. Neck staging is usually determined according to the American Joint Committee on Cancer (AJCC) classification; its inferiority to the number of positive lymph nodes is described in head and neck squamous cell carcinoma^14,15^. The survival significance of the number of positive lymph nodes in salivary gland carcinoma is not frequently evaluated^16,17^. Moreover, no consensus has been reached on the optimal cutoff value of positive lymph node number. Therefore, the current study aimed to determine the effect of positive lymph node number on survival in cN0 MEC of the major salivary gland and to assess the predictors for occult metastasis.

## Patients and methods

### Patient selection

All data were obtained from the surveillance, epidemiology, and end results (SEER) database, which provides information on cancer statistics to reduce the cancer burden among the US population^18^. The profiles of patients who underwent surgical treatment for MEC of the major salivary gland between 1975 and 2019 were reviewed. The inclusion criteria were as follows: the disease was primary; the neck status was cN0; and END was performed. Patients with a history of other cancers or without information on detailed surgical procedures or follow-up were excluded. Demographic characteristics, time to surgery (TTS), histological grade, tumor extension, treatment, and follow-up were extracted and analyzed. Ethical approval was not required because the data are accessible to the public.

### Variable definitions

Pathologic grade was categorized as follows: low for well differentiation, intermediate for moderate differentiation, and high for poor differentiation or undifferentiation^13^. Tumor size was determined based on the Collaborative Stage tumor size (2004–2015), extent of disease (EOD) 10-size (1988–2003), and EOD 4-size (1983–1987). A cN0 neck was confirmed using the Derived EOD classification, Derived AJCC classification, and Derived SEER combined classification. The number of positive lymph nodes was calculated based on Regional nodes examined (1988 +), Regional nodes positive (1988 +), and RX Summ–Scope Reg LN Sur (2003 +). Distant metastasis was determined based on the Derived EOD classification, Derived AJCC classification, and Derived SEER combined classification. Tumor extension was defined as extracapsular and perineural invasion. It was also evaluated based on Collaborative Stage extension (2004–2015), EOD 10-extent (1988–2003), and EOD 4-extent (1983–1987)^14^. TTS was defined as the duration between diagnosis and surgery. END was defined as any case with four or more lymph nodes examined by a pathologist^19^.

### Statistical analysis

The total population was randomly divided into training and test groups with a ratio of 1:1. In the training group, the primary outcome variables were disease-specific survival (DSS) and overall survival (OS). The survival time of DSS was calculated from the date of surgery to the date of cancer-related death or last follow-up. The survival time of OS was calculated from the date of surgery to the date of any-cause death or last follow-up.

Univariate and multivariate Cox analyses were performed to determine the prognostic factors for DSS and OS. A predictive model based on the independent prognostic factors was then constructed for DSS and OS. The two models were evaluated using C-index and calibration curve.

The test group was used to validate the predictive models by C-index and calibration curve.

The second outcome variable was occult metastasis based on the entire population. The chi-square test and logistic regression model were used to detect the independent predictors for occult metastasis. All statistical analyses were performed using R program version 3.4.3. Statistical significance was set at p < 0.05.

## Results

### Baseline data

In total, 3317 patients (men, 1624 [49.0%]; women, 1693 [51.0%]) with a mean age of 55 ± 20 years were included. White race accounted for 75.4% of the entire population, and most (56.0%) of the patients were married at initial diagnosis. Almost 90% of the patients lived in the urban area with a household income greater than 50,000 dollars, and 89.1% of the tumors occurred in the parotid gland. The frequencies of low, intermediate, and high grade disease were 22.2%, 40.1%, and 28.1%, respectively. At the initial treatment, 51 (1.5%) patients had distant metastasis. Of all patients, 6.0% underwent local tumor excision, while the remainder underwent subtotal (41.6%) or total (39.0%) gland resection. Tumor size was larger than 4 cm in 632 (19.1%) patients and no greater than 2 cm in 1402 (42.3%) patients. Tumor extension was present in 922 (27.8%) patients. Surgical treatment was completed within 1 month after diagnosis in 87.5% of the patients, but the TTS was > 3 months in 147 (4.4%) patients (Table 1).

**Table 1:** Baseline data of the entire population, training group, and test group.

Variable	Entire population, N = 3317	Training group, N = 1659	Test group, N = 1658	p*
Age				
≤ 39	788 (23.8%)	399 (24.1)	389 (23.5%)	
40–59	1116 (33.6%)	553 (33.3%)	563 (34.0%)	
60–84	1309 (39.5%)	655 (39.5%)	654 (39.4%)	
85 +	104 (3.1%)	52 (3.1%)	52 (3.1%)	0.975
Sex				
Male	1624 (49.0%)	809 (48.8%)	815 (49.2%)	
Female	1693 (51.0%)	850 (51.2%)	843 (50.8%)	0.822
Race				
White	2501 (75.4%)	1246 (75.1%)	1255 (75.7%)	
Black	396 (11.9%)	202 (12.2%)	194 (11.7%)	
Others	388 (11.7%)	201 (12.1%)	187 (11.3%)	0.723
Marital				
Single	769 (23.2%)	394 (23.7%)	375 (22.6%)	
Married	1857 (56.0%)	932 (56.2%)	925 (55.8%)	
Others	541 (16.3%)	265 (16.0%)	276 (16.6%)	0.723
Income ($)				
≤ 49,999	313 (9.4%)	159 (9.6%)	154 (9.3%)	
50,000–74,999	1784 (53.8%)	864 (52.1%)	920 (55.5%)	
75,000 +	1142 (34.4%)	592 (35.7%)	550 (33.2%)	0.187
Area				
Urban	2914 (87.9%)	1457 (87.8%)	1457 (87.9%)	
Rural	338 (10.2%)	169 (10.2%)	169 (10.2%)	1.000
Site				
Parotid	2955 (89.1%)	1468 (88.5%)	1487 (89.7%)	
Others	315 (9.5%)	167 (10.1%)	148 (8.9%)	0.260
Diagnosis year				
≤ 1999	438 (13.2%)	229 (13.8%)	209 (12.6%)	
2000–2009	1281 (38.6%)	630 (38.0%)	651 (39.3%)	
2010 +	1598 (48.2%)	800 (48.2%)	798 (48.1%)	0.533
Grade				
Low	735 (22.2%)	369 (22.2%)	366 (22.1%)	
Intermediate	1329 (40.1%)	650 (39.2%)	679 (41.0%)	
High	447 (28.1%)	225 (28.5%)	222 (27.7%)	0.978
Distant metastasis				
M0	2718 (81.9%)	1359 (81.9%)	1359 (82.0%)	
M1	51 (1.5%)	27 (1.6%)	24 (1.4%)	0.677
Operation				
Local excision	199 (6.0%)	105 (6.3%)	94 (5.7%)	
Subtotal gland excision	1379 (41.6%)	692 (41.7%)	687 (41.4%)	
Total gland excision	1294 (39.0%)	627 (37.8%)	667 (40.2%)	0.436
Positive lymph node number				
0	2503 (75.5%)	1247 (75.2%)	1256 (75.8%)	
1	369 (11.1%)	193 (11.6%)	176 (10.6%)	
2	119 (3.6%)	60 (3.6%)	59 (3.6%)	
3–5	145 (4.4%)	75 (4.5%)	70 (4.2%)	
6 +	181 (5.5%)	84 (5.1%)	97 (5.9%)	0.749
Tumor extension				
No	2043 (61.6%)	1009 (60.8%)	1034 (62.4%)	
Yes	922 (27.8%)	477 (28.8%)	445 (26.8%)	0.237
Tumor size (cm)				
≤ 2	1402 (42.3%)	700 (42.2%)	702 (42.3%)	
2–4	1193 (36.0%)	639 (38.5%)	644 (38.8%)	
> 4	632 (19.1%)	320 (19.3%)	312 (18.8%)	0.707
Time to surgery (month)				
≤ 1	2902 (87.5%)	1457 (87.8%)	1445 (87.2%)	
1–2	256 (7.7%)	125 (7.5%)	131 (7.9%)	
3 +	147 (4.4%)	73 (4.4%)	74 (4.5%)	0.910
Radiotherapy				
No	1690 (50.9%)	841 (50.7%)	849 (51.2%)	
Yes	1580 (47.6%)	796 (48.0%)	784 (47.3%)	0.725
Chemotherapy				
No	3129 (94.3%)	1557 (93.9%)	1572 (94.8%)	
Yes	188 (5.7%)	102 (6.1%)	86 (5.2%)	0.231

*Comparison between training group and test group.

Occult metastasis occurred in 814 patients, with a rate of 24.5%. In these 814 patients, 369 (11.1%) had one positive lymph node, 119 (3.6%) had two positive lymph nodes, 145 (4.5%) had three to five positive lymph nodes, and 181 (5.5%) had six or more positive lymph nodes.

### Predictor for occult metastasis

Occult metastasis developed in 48.1% of the patients aged 85 + years; the rate was apparently higher than 15.5% in patients aged ≤ 39 years (p < 0.001). The women had an occult metastasis rate of 17.3%, which was statistically lower than that (32.1%) in men. Positive lymph node was noted in 26.3%, 19.2%, and 19.3% of the white, black, and other race patients, respectively; the difference was statistically significant (p < 0.001). Occult metastasis rate showed apparent difference in patients with different marital statuses (p = 0.016). Living in the rural area was associated with higher possibility of occult metastasis than living in the urban area (30.5% vs. 23.5%, p = 0.005). Among the tumors with different grade, high grade disease had the highest rate of occult metastasis of 54.2% (p < 0.001). The presence of tumor extension was associated with increased possibility of occult metastasis (46.7% vs. 14.6%, p < 0.001). A tumor greater than 4 cm in size had a rate of 49.1% of occult metastasis, which was higher than that in smaller tumors (p < 0.001). No statistical relationships were detected between occult metastasis and household income, and primary site (all p > 0.05, Table 2).

**Table 2:** Univariate and logistic regression analysis of predictors for occult metastasis in total population.

Variable	Occult metastasis		p
	No (n = 2503)	Yes (n = 814)	
Age			
≤ 39	666 (94.5%)	122 (15.5%)	
40–59	874 (78.3%)	242 (21.7%)	
60–84	909 (69.4%)	400 (30.6%)	
85 +	54 (51.9%)	50 (48.1%)	< 0.001
Sex			
Male	1103 (79.2%)	521 (20.8%)	
Female	1400 (82.7%)	293 (17.3%)	< 0.001
Race			
White	1842 (73.7%)	659 (26.3%)	
Black	320 (80.8%)	76 (19.2%)	
Others	313 (80.7%)	75 (19.3%)	< 0.001
Marital			
Single	602 (78.3%)	167 (21.7%)	
Married	1394 (75.1%)	463 (24.9%)	
Others	386 (71.3%)	155 (28.7%)	0.016
Income ($)			
≤ 49,999	226 (72.2%)	87 (27.8%)	
50,000–74,999	1372 (76.9%)	412 (23.1%)	
75,000 +	854 (74.8%)	288 (25.2%)	0.134
Area			
Urban	2228 (76.5%)	686 (23.5%)	
Rural	235 (69.5%)	103 (30.5%)	0.005
Site			
Parotid	2243 (75.9%)	712 (24.1%)	
Others	234 (65.5%)	81 (34.5%)	0.524
Diagnosis year			
≤ 1999	303 (69.2%)	135 (30.8%)	
2000–2009	914 (71.4%)	367 (28.6%)	
2010 +	1286 (80.5%)	312 (19.5%)	< 0.001
Grade			
Low	683 (92.9%)	52 (7.1%)	
Intermediate	1146 (86.2%)	183 (13.8%)	
High	426 (45.8%)	505 (54.2%)	< 0.001
Tumor extension			
No	1745 (85.4%)	298 (14.6%)	
Yes	491 (53.3%)	431 (46.7%)	< 0.001
Tumor size (cm)			
≤ 2	1280 (91.3%)	122 (8.7%)	
2–4	963 (80.7%)	230 (19.3%)	
> 4	322 (50.9%)	310 (49.1%)	< 0.001

In logistic regression analysis, age greater than 85 years (p = 0.028, 2.005 [1.076–3.737]), intermediate (p = 0.005, 2.252 [1.280–3.960]), and high (p < 0.001, 10.752[5.945–19.854]) grades, tumor extension (p < 0.001, 2.514 [1.973–3.204]), and tumor size > 2 cm were independently associated with the risk of occult metastasis (Table 3).

**Table 3:** Logistic regression analysis of predictors for occult metastasis in total population.

Variable	p	OR [95% CI]
Age		
≤ 39		Ref
40–59	0.592	1.106 [0.766–1.598]
60–84	0.247	1.247 [0.858–1.813]
85 +	0.028	2.005 [1.076–3.737]
Sex (female vs male)	0.056	0.784 [0.612–1.006]
Race		
White		Ref
Black	0.479	1.154 [0.776–1.715]
Others	0.712	0.927 [0.620–1.386]
Marital		
Single		Ref
Married	0.817	0.962 [0.696–1.331]
Others	0.627	0.905 [0.606–1.353]
Area (rural vs urban)	0.321	1.202 [0.836–1.726]
Diagnosis year		
≤ 1999		Ref
2000–2009	0.772	0.949 [0.665–1.354]
2010 +	0.184	0.777 [0.536–1.127]
Grade		
Low		Ref
Intermediate	0.005	2.252 [1.280–3.960]
High	< 0.001	10.752 [5.945–19.854]
Tumor extension	< 0.001	2.514 [1.973–3.204]
Tumor size (cm)		
≤ 2		Ref
2–4	0.013	1.437 [1.103–1.968]
> 4	< 0.001	1.908 [1.488–2.537]

### Predictive model for DSS

In the training group, the mean follow-up time was 95 ± 83 months, and the 5- and 10-year DSS rates were 86% (95% confidence interval CI 84–88%) and 82% (95% CI 80–84%), respectively. In univariate Cox analysis, compared with patients without occult metastasis, positive lymph node was associated with decreased disease control. Other potential predictors for DSS included age, sex, race, marital status, primary site, diagnosis year, grade, distant metastasis, operation type, tumor extension, tumor size, and radiotherapy. In multivariate Cox analysis, age, grade, distant metastasis, number of positive lymph nodes, tumor extension, and tumor size were independent prognostic factors. One positive lymph node (p = 0.452, 1.335 [0.629–2.833]) did not pose additional compromise to DSS compared with no lymph node metastasis. The negative effect began when two positive lymph nodes were present, and disease control was poorer along with the number of positive lymph nodes (Table 4).

**Table 4:** Univariate and multivariate Cox analysis of predictors for disease specific survival (DSS) and overall survival (OS) in training group.

Variable	DSS			OS		
	Univariate	Multivariate		Univariate	Multivariate	
Age						
≤ 39			Ref			Ref
40–59	< 0.001	0.295	1.509 [0.699–3.259]	< 0.001	0.002	2.598 [1.412–4.781]
60–84	< 0.001	0.007	2.816 [1.334–5.946]	< 0.001	< 0.001	6.850 [3.808–12.320]
85 +	< 0.001	< 0.001	7.127 [2.848–17.830]	< 0.001	< 0.001	25.178 [12.235–51.814]
Sex						
Male			Ref			Ref
Female	< 0.001	0.220	0.771 [0.508–1.168]	< 0.001	0.307	0.859 [0.641–1.150]
Race						
White			Ref			Ref
Black	0.163	0.821	0.927 [0.478–1.795]	0.142	0.893	0.968 [0.605–1.549]
Others	0.010	0.530	0.801 [0.401–1.601]	0.001	0.446	0.823 [0.499–1.358]
Marital						
Single			Ref			Ref
Married	0.010	0.713	0.904 [0.527–1.551]	< 0.001	0.698	0.922 [0.613–1.387]
Others	< 0.001	0.260	0.694 [0.368–1.309]	< 0.001	0.841	0.954 [0.600–1.517]
Income ($)						
≤ 49,999						
50,000–74,999	0.259			0.059		
75,000 +	0.534			0.124		
Area						
Urban						Ref
Rural	0.083			0.022	0.211	1.264[0.875–1.825]
Site						
Parotid			Ref			Ref
Others	0.002	0.560	1.208 [0.641–2.275]	0.004	0.591	1.132 [0.719–1.782]
Diagnosis year						
≤ 1999			Ref			Ref
2000–2009	0.729	0.485	1.351 [0.581–3.141]	0.434	0.747	1.098 [0.624–1.933]
2010 +	0.007	0.533	0.751 [0.305–1.847]	0.004	0.235	0.686 [0.368–1.278]
Grade						
Low			Ref			Ref
Intermediate	0.033	0.226	3.512 [0.460–26.794]	0.620	0.077	0.595 [0.335–1.058]
High	< 0.001	0.018	11.145 [1.542–86.728]	< 0.001	0.010	2.023 [1.077–5.654]
Distant metastasis						
M0			Ref			Ref
M1	< 0.001	0.034	2.386 [1.067–5.337]	< 0.001	0.045	1.983 [1.015–3.872]
Operation						
Local excision			Ref			Ref
Subtotal gland excision	0.812	0.913	1.056 [0.397–2.807]	0.430	0.453	1.296 [0.658–2.550]
Total gland excision	0.013	0.350	1.582 [0.605–4.136]	0.004	0.132	1.669 [0.857–3.249]
Positive lymph node number						
0			Ref			Ref
1	< 0.001	0.452	1.335 [0.629–2.833]	< 0.001	0.926	1.028 [0.580–1.820]
2	< 0.001	0.002	2.176 1.321–3.585]	< 0.001	0.082	1.399 [0.958–2.043]
3–5	< 0.001	0.001	2.561 [1.437–4.565]	< 0.001	0.006	1.896 [1.204–2.988]
6 +	< 0.001	< 0.001	3.178 [1.830–5.519]	< 0.001	0.001	2.176 [1.388–3.412]
Tumor extension						
No			Ref			Ref
Yes	< 0.001	0.014	2.004 [1.654–3.541]	< 0.001	0.398	1.140 [0.841–1.544]
Tumor size (cm)						
≤ 2			Ref			Ref
2–4		0.005	1.883 [1.482–2.554]		0.017	1.548 [1.322–1.894]
> 4	< 0.001	< 0.001	2.835 [2.014–4.307]	< 0.001	< 0.001	2.696 [2.037–3.569]
Radiotherapy						
Yes			Ref			Ref
No	< 0.001	0.669	1.113 [0.682–1.816]	< 0.001	0.467	0.887 [0.643–1.224]

**Figure 1:**
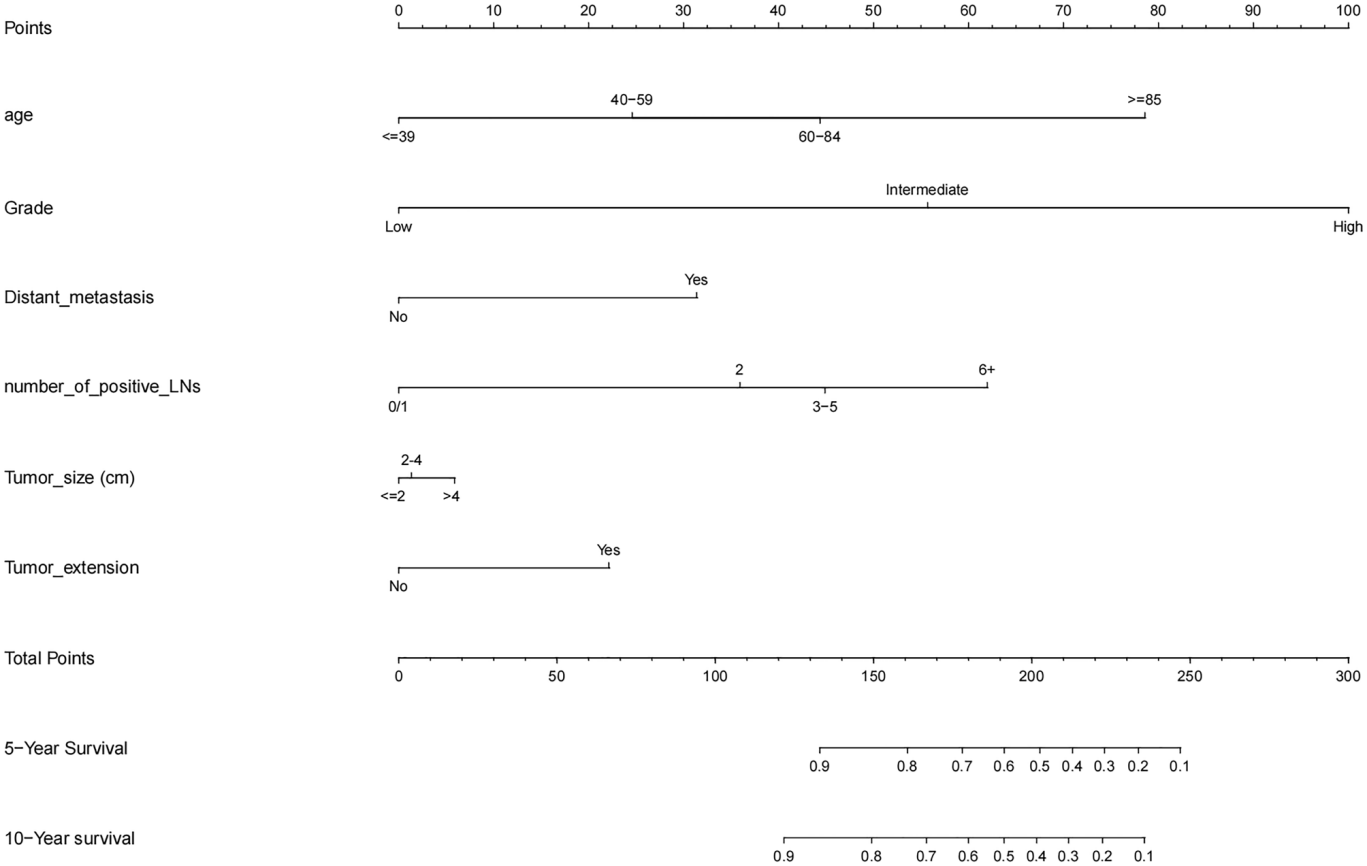
Nomogram for predicting the disease specific survival.

**Figure 2:**
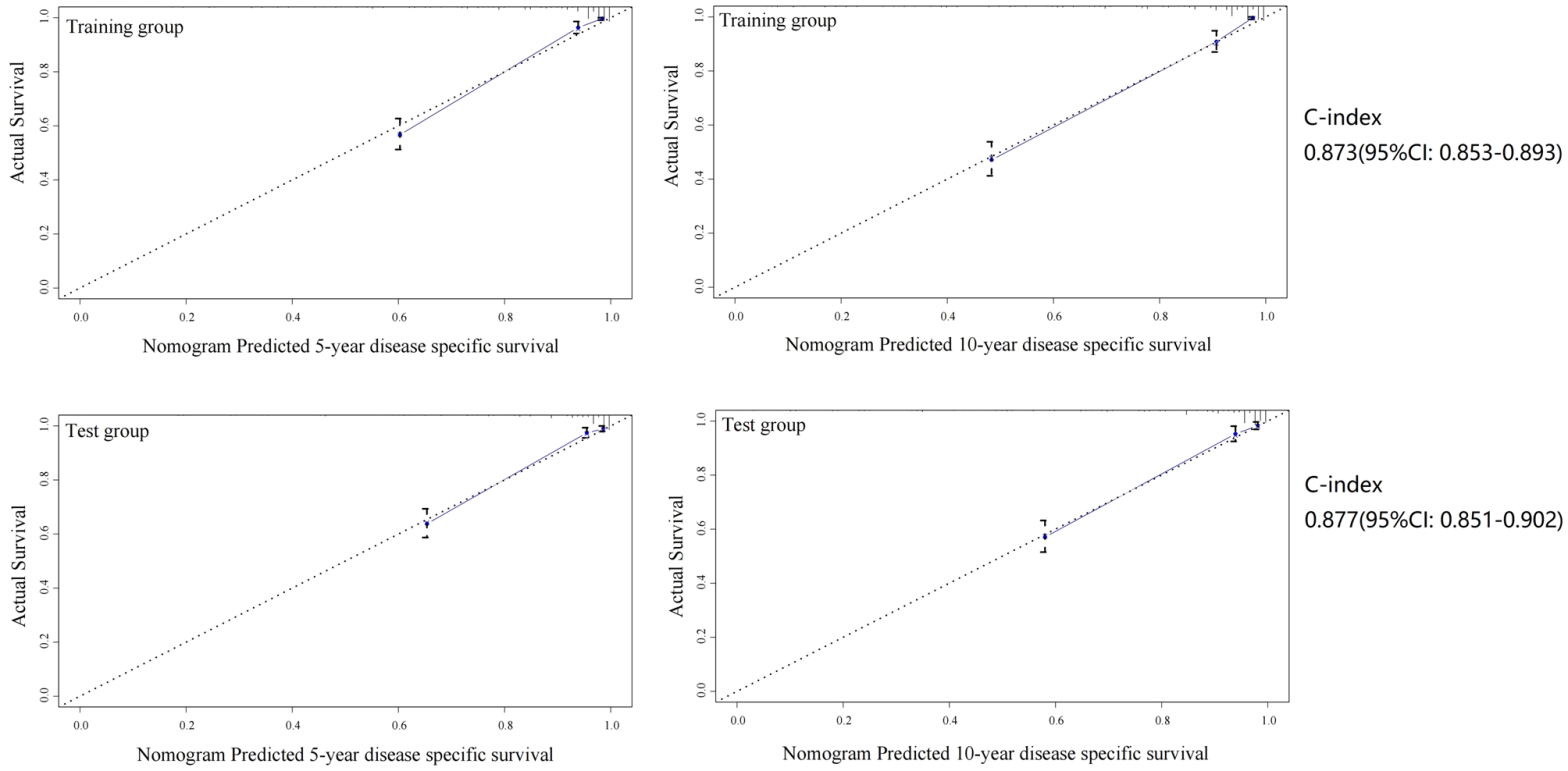
Nomogram predicted 5- and 10- year disease specific survival rates in training and test groups.

A predictive model for DSS was constructed using the following seven factors: age, grade, distant metastasis, number of positive lymph nodes, tumor extension, and tumor size (Fig. 1). The model had a C-index of 0.873 (95% CI 0.853–0.893) (Fig. 2).

### Predictive model for OS

In the training group, the 5- and 10-year OS rates were 76% (95% CI 74–78%) and 65% (95% CI 63–67%), respectively. In univariate Cox analysis, compared with patients without occult metastasis, positive lymph node was associated with increased overall death. Other potential predictors for DSS included age, sex, race, area, marital status, primary site, diagnosis year, grade, distant metastasis, operation type, tumor extension, tumor size, and radiotherapy. In multivariate Cox analysis, age, grade, distant metastasis, number of positive lymph nodes, and tumor size were independent prognostic factors. One (p = 0.926, 1.028 [0.580–1.820]) or two (p = 0.082, 1.399 [0.958–2.043]) positive lymph nodes did not pose additional compromise to OS compared with no lymph node metastasis. The negative effect began when at least three positive lymph nodes were present, and overall death was greater along with the number of positive lymph nodes (Table 4).

**Figure 3:**
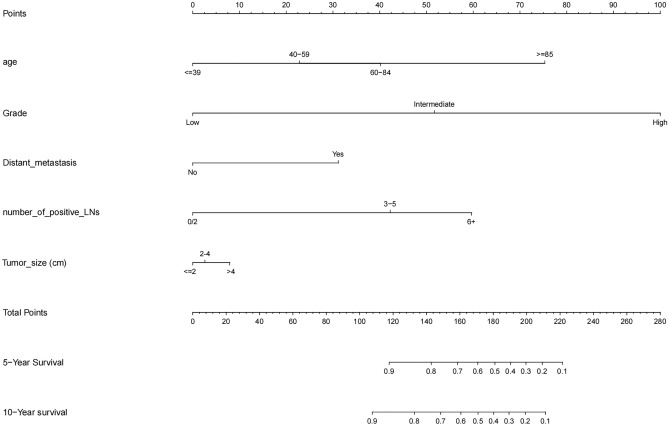
Nomogram for predicting the overall survival.

**Figure 4:**
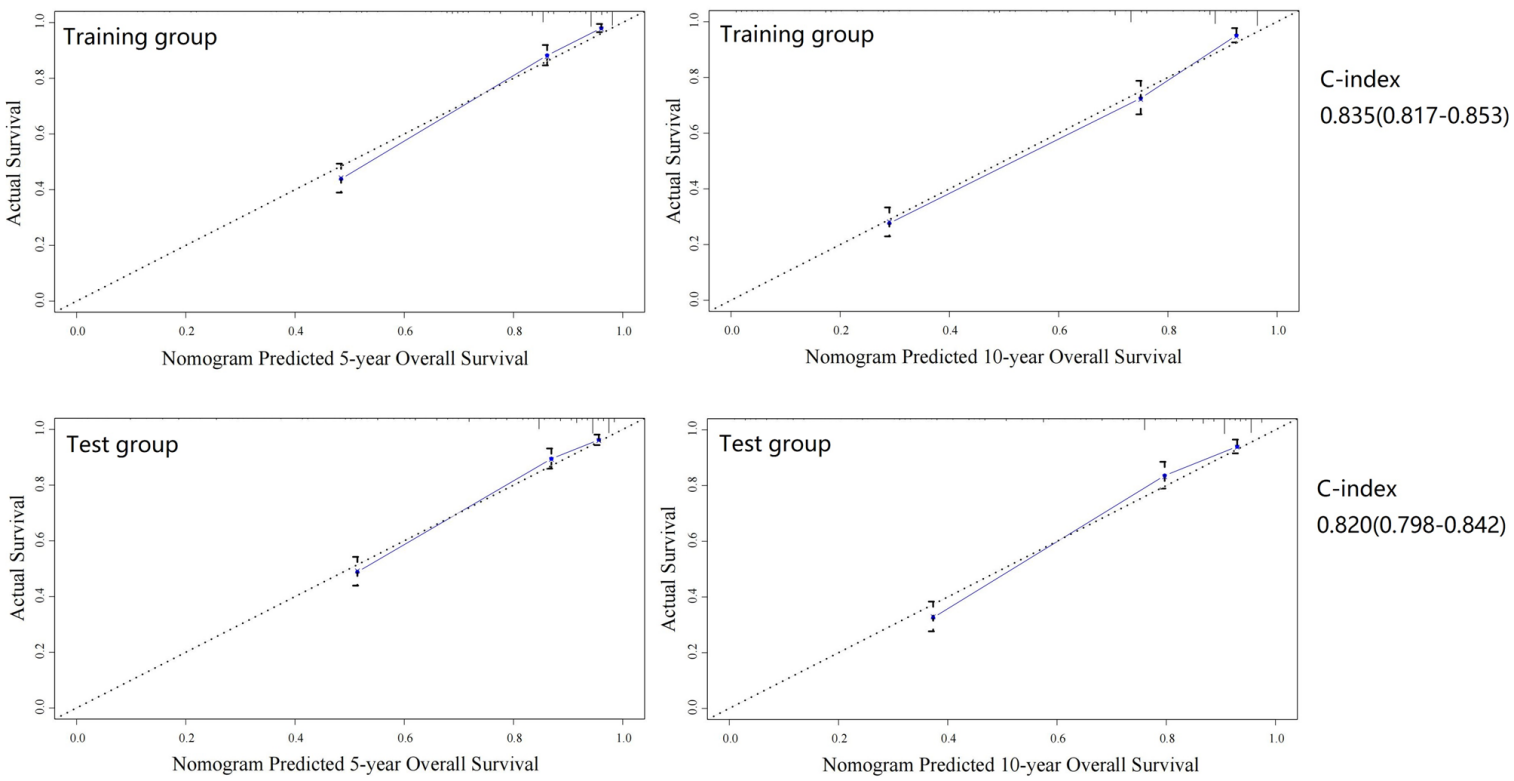
Nomogram predicted 5- and 10- year overall survival rates in training and test groups.

A predictive model for OS was constructed using the following six factors: age, grade, distant metastasis, number of positive lymph nodes, and tumor size (Fig. 3). The model had a C-index of 0.835 (95% CI 0.817–0.853) (Fig. 4).

### Validation of the two models

In the test group, the mean follow-up time was 96 ± 81 months, and the 5- and 10-year DSS rates were 87% (95% CI 85–89%) and 85% (95% CI 83–87%), respectively. The 5- and 10-year OS rates were 76% (95% CI 74–78%) and 68% (95% CI 66–70%), respectively. Based on the two models, the C-indexes calculated in the test group were 0.877 (95% CI 0.851–0.902) and 0.820 (95% CI 0.798–0.842), respectively (Figs. 2 and 4).

## Discussion

The most important finding in the current study is that the number of positive lymph nodes is statistically associated with survival in cN0 major salivary gland MEC. Adverse effects begins when at least two positive lymph nodes and at least three positive lymph nodes were present for DSS and OS, respectively. The two prognostic models have high accuracy in predicting oncology outcome. Another interesting finding is that age greater than 85 years is associated with higher possibility of occult metastasis.

Lymph node status is usually one of the most important prognostic factors in solid cancer. The current neck staging for salivary gland carcinoma is entirely based on the 8th AJCC system calculated from head and neck squamous cell carcinoma^20^, which takes lymph node size, laterality, and extranodal extension (ENE) into consideration. However, significant differences exist with regard to biologic behavior, treatment modalities, and oncologic outcome between the two entities. The application of the neck staging system in forecasting the results of salivary gland carcinoma is questioned.

The prognostic significance of the number of positive lymph nodes is rarely assessed. Aro et al.^21^ might be the first to evaluate the effect of quantitative lymph node burden in salivary gland carcinoma. Based on 4520 patients who had undergone END, an increasing number of positive lymph nodes were strongly associated with worse survival without plateau. Moreover, the risk of overall death increased more rapidly up to four lymph nodes and was more gradual for additional lymph nodes > 4. After accounting for the number of metastatic lymph nodes, ENE and lymph node size had no effect on OS. Moreover, a new lymph node staging system was proposed according to the number of positive lymph nodes (0 vs. 1–2 vs. 3–21 vs. 22 +) and exhibited greater concordance than the official one. However, as the authors commented, owing to their specific inclusion criteria, their results were only suitable to patients who required neck dissection due to a cN + neck or high risk features. In a study by Lombardi et al.^17^, ENE was not associated with worse OS, and three novel N-classifications were developed based on the number of positive lymph nodes (0 vs. 1–3 vs. 4 +) and/or their maximum size (< 2 cm vs. ≥ 2 cm). Compared with the current N classification, better accuracy in OS stratification was noted in the three models confirmed using the superior Akaike information criterion, Bayesian information criterion, and Nagelkerke pseudo-R^2^. Qian et al.^16^ divided 3046 pN + patients into two groups with a cutoff value of 4 for positive lymph nodes. The number of positive lymph nodes greater than 4 carried additional 80% risk of overall death compared with that of positive lymph nodes less than 4. The same team analyzed the outcomes of 66 patients with salivary duct carcinoma and reported that eight or more positive lymph nodes rather than N2-3 stage and ENE was an independent predictor for OS^22^. Although the cutoff value was inconsistent, all these studies confirmed that the number of positive lymph nodes was superior to the current N stage method in distinguishing different populations. However, the pathology types of salivary gland carcinoma are complex; whether the conclusion could be applied for some other types of salivary gland carcinomas remained unclear.

In the current study, compared with pN0 disease, no additional compromise to DSS and OS was required if only one positive lymph node and if no more than two positive lymph nodes, respectively, were present. The presence of only lymph node can typically decrease the survival by up to 50% in head and neck squamous cell carcinoma^23^. Our finding partially reflected the inherent difference between MEC and head and neck squamous cell carcinoma. In previous similar studies, pN0 patients had statistically better OS than patients with one or two positive lymph nodes^17,21^. The difference might be attributed to the pathologic complexity of salivary gland carcinoma that has unique features. MEC is graded into well, moderate, poor, and undifferentiated. A well-grade type disease tends to grow slowly and has low possibility of occult metastasis, but undifferentiated disease is likely to invade the surrounding tissue aggressively^24^.

Two prognostic models were constructed for DSS and OS with high C-index; they could provide individualized follow-up strategies for patients. A few authors developed similar models for parotid gland carcinoma^25^, but their C-indexes range from 0.608 to 0.765, which is lower than ours. The difference might be attributed to differences in epidemiology, treatment modalities, and analyzed variables. Lan et al.^26^ also introduced a predictive model for DSS and OS, and the C-index is as high as 0.871. However, the enrollment of non-surgical patients prevented its clinical application. The current model, which was endowed with high reliability, was formulated based on independent prognostic factors. Conversely, traditional N stage was replaced by the number of positive lymph nodes, which offered increased accuracy.

Predictors for occult metastasis in MEC are extensively assessed; the commonly accepted factors include advanced stage, and adverse pathologic features^3–5^. However, the effect of age on occult metastasis is rarely reported. Fussey et al.^1^ noted age ≥ 60 years is associated with additional 1.5-fold risk of lymph node metastasis. The current study found that patients aged 85 + years have statistically higher rate of occult metastasis than the younger patients. The underlying mechanism might be that long duration of undetected tumors owing to the absence of symptoms gave more chance of occult metastasis. The adverse impact of pathologic grade on occult metastasis was found to be significant. High-grade tumors exhibited a 54.2% likelihood of lymph node metastasis, which was markedly higher compared to low/intermediate grade tumors, a correlation supported by previous studies^5,6^. Interestingly, it was observed that tumors treated after 2010 displayed a reduced risk of occult metastasis in comparison to those treated earlier (19.5% vs. 28.5% vs. 30.8%, p < 0.001). This may be attributed to the examination methods employed prior to surgery, where modern and improved techniques enabled the detection of smaller metastases, thereby reducing their likelihood to remain occult compared to older techniques.

This study has four main limitations. First, this was a retrospective study, which decreased the statistical power. Second, we did not consider the margin status, which might have affected survival outcomes. Third, we did not assess the influence of tumor stage, which was determined according to the 6th, 7th, and 8th editions of the AJCC classification. Fourth, data regarding metastatic levels was not available and could not be assessed.

In conclusion, for cN0 major salivary gland MEC, elective neck dissection is recommended in cases of high grade tumors. The presence of positive lymph nodes is statistically linked to survival outcomes, and the existing prognostic model has the potential to offer personalized follow-up strategies for patients with a high degree of accuracy.

## Data Availability

All data generated or analyzed during this study are included in this published article. And the primary data could be achieved from the corresponding author.
